# Detecting and differentiating neurotransmitters using ultraviolet plasmonic engineered native fluorescence†

**DOI:** 10.1039/d3ra05405e

**Published:** 2023-11-07

**Authors:** Ji-Young Lee, Mohammad Mohammadi, Yunshan Wang

**Affiliations:** a Department of Chemical Engineering, University of Utah Salt Lake City 84112 USA yunshan.wang@utah.edu

## Abstract

Detecting neurotransmitters with high sensitivity and selectivity is important to understand their roles in biological functions. Current detection methods for neurotransmitters suffer from poor sensitivity or selectivity. In this article, we propose ultraviolet (UV) plasmonic engineered native fluorescence as a new sensing mechanism to detect neurotransmitters with high sensitivity and selectivity. We measured the native fluorescence of three monoamine neurotransmitters, dopamine (DA), norepinephrine (NE), and 3,4-dihydroxyphenylacetic acid (DOPAC). The average net enhancement and total photon yield enhancement on an aluminum hole array with 300 nm hole spacing substrate were found to be 50× and 60×, for the three molecules. We also observed a 1.5–1.7× reduction in the dominant photon bleaching rate on an aluminum hole array compared to an aluminum-thin film substrate. The photobleaching rates of the native fluorescence of DA, NE and DOPAC were found to be highly sensitive to their molecular structures and can be further engineered by UV plasmonic substrates. The differences in the photobleaching rates for DA and NE were 2× and 1.6× larger on an aluminum thin film and an aluminum hole array than on a silicon substrate. As a proof-of-concept experiment, we mixed DA with NE at different concentration ratios and measured the average photobleaching rates of the mixture. We found that the average photobleaching rate is proportional to the concentration of NE in the mixture. Our findings demonstrate the potential of UV plasmonic engineered native fluorescence to achieve sensitive and selective detection of neurotransmitters.

## Abstract 2

Detecting neurotransmitters with high sensitivity and selectivity is important to understand their roles in biological functions.

## Graphical abstract

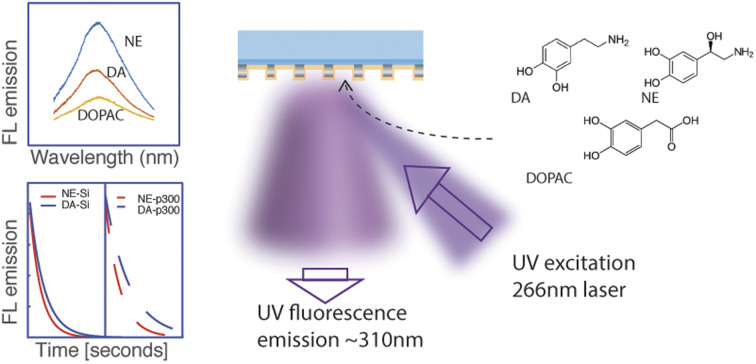

## Introduction

Monoamine neurotransmitters (MANTs), such as dopamine (DA), norepinephrine (NE), and 3,4-dihydroxyphenylacetic acid (DOPAC), play a crucial role in the endocrine and central nervous systems.^1–3^ The fluctuation levels of MANTs are the result of particular neurological or immunological diseases, such as Parkinson's disease, human immunodeficiency virus infection, and schizophrenia.^4,5^ The physiological concentration of MANT in the bodily fluid is low, ranging from hundreds of picomolar (pM) to a low nanomolar (nM) range.^6^ MANTs and their derivatives with similar molecular structures but different functions usually coexist in bodily fluids. Current technologies for analysis of MANTs include liquid chromatography-mass spectroscopy (LC-MS),^7^ fast-scan cyclic voltammetry (FSCV),^8^ and nanomaterial-based biosensors.^9^ LC-MS provides high sensitivity and selectivity; however, it requires substantial sample preparation, which can result in sample loss. FSCV requires minimal sample preparation and can detect MANTs *in vivo*. However, this technique is not selective as different molecules can have overlapping redox potentials.^5^ Nanomaterial-based biosensors require either aptamers,^10–13^ antibodies, enzymatic or chemical reactions,^14–19^ for selective detection. However, the selection of highly specific aptamers or antibodies can be challenging and time-consuming and the nonspecific binding to interfering molecules still occurs even with carefully selected aptamers.^11,16^ Enzymatic or chemical reactions, although specific, suffer from poor sensitivity.^18^ Cell-based neurotransmitter fluorescent-engineered reporters (CNiFERs) have demonstrated nanomolar concentration detection of DA and NE with high specificity.^20^ However, implantation of reporters to target brain regions might interfere with the natural signalling process. A new sensing mechanism that can directly detect and differentiate neurotransmitters with high sensitivity and specificity without reporters, enzymatic or chemical reaction, is needed.

In this article, we propose the ultraviolet (UV) plasmonic engineered native fluorescence of neurotransmitters as a new sensing mechanism to achieve highly sensitive and specific detection of neurotransmitters. Intrinsic fluorescence-based sensors have been used in environmental monitoring, cell imaging, and analytical chemistry.^21–26^ For example, protein arrays were detected using a time-resolved UV native fluorescence,^26^ and multiphoton excitation of native protein fluorescence has been shown to discriminate ligands with different binding affinities.^27^ Regarding neurotransmitters, dopamine dynamics have been imaged in a slice of mouse brain without dye labeling using two-photon excitation at visible wavelengths and non-epi fluorescence detection at near-UV range.^28^ However, the low quantum yield and unstable nature of the intrinsic fluorescence of biomolecules are limitations of UV intrinsic fluorescence-based biosensors. UV plasmonic enhanced fluorescence improves the sensitivity and limits of detection of UV biosensors.^29–43^ In our previous study, an aluminium (Al) hole array achieved a net enhancement of 50× for tryptophan.^39^ In this study, we demonstrated that a similar enhancement factor was achieved for DA, NE and DOPAC on an Al hole array. We also observed that DA, NE and DOPAC have distinct photobleaching rates under UV illumination and the differences in their photobleaching rates are enlarged on an Al thin film and an Al hole array. We thus propose to use the UV plasmonic engineered native fluorescence photobleaching rates as a new mechanism for differentiating neurotransmitters without the need for aptamer, antibody, chemical, or enzymatic reactions. Our findings suggest that the UV plasmonic engineered native fluorescence has the potential to achieve highly sensitive and selective detection of neurotransmitters without recognition probes or reactions.

## Experimental

### Materials

Materials were purchased from Sigma-Aldrich Co. (St. Louis, MO). Non-functionalized polystyrene nanospheres (PS, nominal diameter 300 nm) were obtained from Bangs Laboratories. The 10% nanosphere suspension was prepared in a solution containing ethanol and water (ethanol : H_2_O = 2 : 3 by volume). A 2 inch silicon wafer (with a 1.7 nm-thick silicon dioxide layer) was cut into quarters and used as a substrate. Dopamine (DA), 3,4-dihydroxyphenylacetic acid (DOPAC) and norepinephrine (NE) were dissolved in a 0.25 wt% aqueous solution of polyvinyl alcohol (PVA) to 1 mM, respectively.

### Fabrication methods

**Fig. 1:**
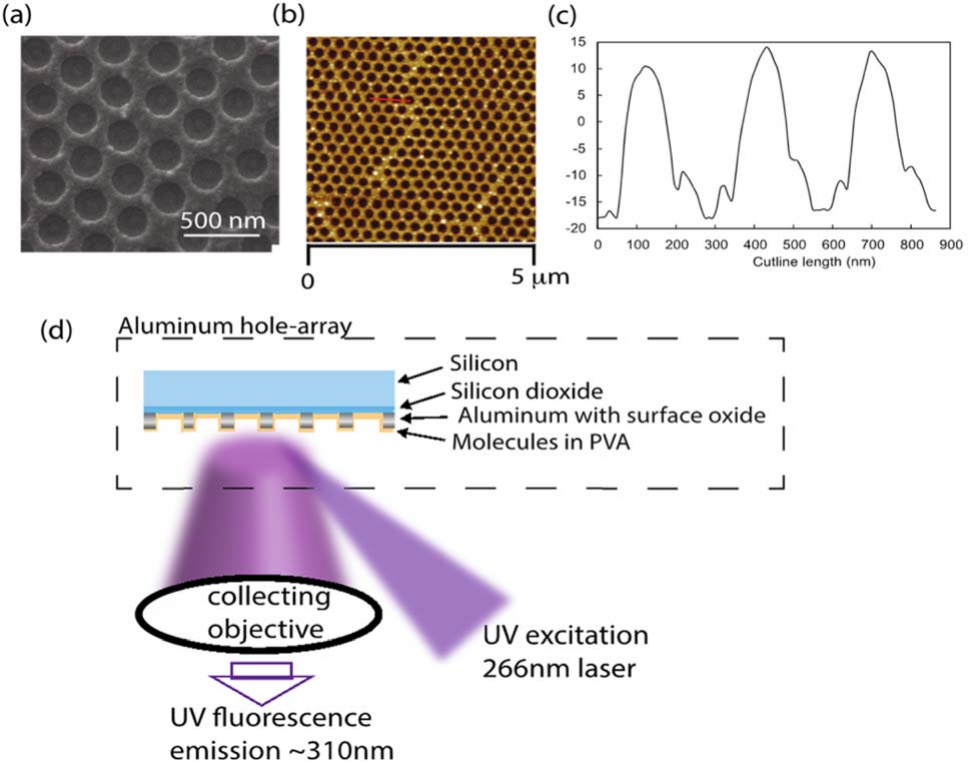
(a) An SEM image and (b) an AFM image of a hole array with 300 nm hole spacing. (c) The height profile for a red line is drawn in the AFM image. (d) Schematic of the experimental setup with an Al hole array substrate. The Al hole array is on a silicon substrate with a thin layer of silicon dioxide (∼1.7 nm), the Al layer is 30 nm in thickness and has a native oxide layer on top (∼4 nm). The molecules are dissolved in a PVA solution and spin-coated on the hole array surface. The native fluorescence of molecules is excited by a 266 nm CW laser and the emitted fluorescence is captured by a CCD camera.

We measured the photobleaching rates of neurotransmitters on three different substrates-an Al hole array with a hole spacing of 300 nm (P300), an Al 30 nm thin film and a silicon substrate. The P300 Al hole array and the Al 30 nm thin film were fabricated based on a previously reported procedure.^38^ Briefly, polystyrene nanospheres (PS) were spin-coated for 70 seconds at 800 rpm onto silicon wafers. PS was etched on the substrate (using the Oxford Plasmalab 80 Plus) until it reached a diameter of 216 (±6) nm, which is shown in ESI Fig. S1.† Following the etching process, a 30 nm-thick layer of Al was deposited over the etched PS by electron beam evaporation (Denton SJ20C). Residual PS particles were removed by ultrasonication in isopropyl alcohol (IPA) for 1 hour. As a result, an Al hole array with a hole size of 215 (±5) nm and a hole spacing of 300 nm was fabricated. The 30 nm-thick Al film was deposited by electron beam evaporation on a silicon wafer. Neurotransmitters were dissolved in 0.25 wt% PVA solution and then spin-coated for 30 seconds at 3000 rpm on the surface of the substrates. The thickness of the PVA film with neurotransmitters after evaporation is about 10 nm.^44^ SEM and AFM images of the Al hole array are shown in Fig. 1a and b. The thickness of the Al in the hole array sample was estimated to be 30 nm using an AFM height profile in Fig. 1c.

### Spectroscopy

The absorption and emission spectrum of the neurotransmitters were measured in a quartz cuvette using a UV-vis spectrometer (Hitachi F-700 Fluorescence Spectrophotometer). The quantum yield of the native fluorescence of the neurotransmitters was measured according to a published method.^45^ The quantum yield of tryptophan in water was used as the reference sample. A detailed description of the method to measure the quantum yields of the neurotransmitters can be found in the ESI.†

Fig. 1d shows the experimental setup to measure the native fluorescence of neurotransmitters on a substrate. A high-intensity 266 nm UV continuous-wave (CW) laser (CryLas, Germany) with a 12 mW power was focused by a plano-convex lens (focal length 100 mm) to a spot size of 60 by 60 μm and impinges on a sample stage at a 60° incident angle. The samples were neurotransmitters spin-coated on different substrates. Upon illumination by the 266 nm CW laser, the native fluorescence of neurotransmitters was excited and collected by a UV objective (focal length 25 mm, F/2.8, UV2528, Universe Kogaku America). The fluorescence emission passed through a long-pass filter (LP02-266RU-25, SEMRock), a cylindrical lens (focal length 100 mm) and entered into a Horiba iHR550 imaging spectrometer with a UV enhanced CCD camera. Fluorescence photobleaching series were collected using an 0.5 second integration time for 90 seconds.

## Results and discussion

**Fig. 2:**
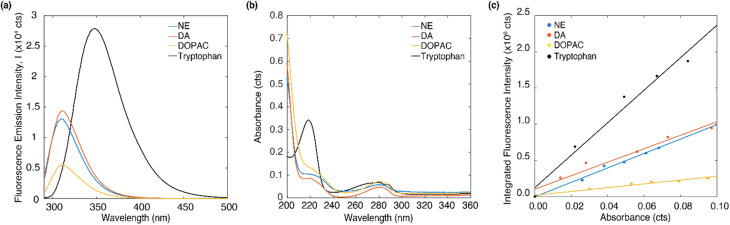
(a) The fluorescence emission spectrum and (b) absorption spectrum of DA, DOPAC, NE and tryptophan dissolved in DI water of 4 μM concentration. (c) Integrated fluorescence intensity data *versus* absorbance for different concentrations of DA, DOPAC, NE dissolved in DI water (0, 10, 20, 30, 40, 50 μM) and linear fitting curve.

The emission and absorption spectra of DA, NE, DOPAC and tryptophan were measured in DI water at 4 μM concentration (Fig. 2a and b). Upon excitation at 266 nm, all three molecules, DA, NE, and DOPAC, exhibited two emission peaks around 297 nm and 314 nm, while tryptophan exhibited two emission peaks at 297 nm and 349 nm. Absorption peaks around 220 and 280 nm were observed for all four molecules, while tryptophan shows a much higher absorption peak at 220 nm. Furthermore, at an excitation wavelength of 266 nm, tryptophan exhibited the highest absorption intensity, followed by DOPAC and NE, while DA showed a similar absorption intensity as NE. Notably, the absorption/emission spectra of DOPAC differed from those of DA or NE in terms of the relative amplitudes of their respective peaks. However, the absorption/emission spectra of DA and NE were highly similar due to the similarity of their molecular structures, making it challenging to differentiate DA from NE based on their absorption/emission spectra alone.

In order to understand what contributes to the relative absorption/emission intensity of neurotransmitters, we measured the quantum yield of DA, DOPAC and NE. The quantum yield can be obtained by taking the ratio of integrated fluorescence intensity to the optical density at excitation wavelength and comparing that with a reference molecule. Detailed description of the procedure to determine quantum yield can be found in the ESI.†Fig. 2c plots the integrated fluorescence intensity of DA, DOPAC and NE at 10, 20, 30 40 and 50 μM *versus* the absorbance at each concentration. The concentration range of 0 to 50 μM was chosen to minimize re-absorption effects. The quantum yield of DA, DOPAC and NE in DI water were determined to be 5.93 ± 0.8%, 1.67 ± 0.21% and 6.34 ± 0.62%. Quantum yield is the proportion of absorbed photons that are being emitted as fluorescence. The higher emission intensity of NE compared to DA was attributed to its higher quantum yield. Furthermore, tryptophan has a higher emission intensity compared to the neurotransmitters due to its larger quantum yield (13%).^45^

**Fig. 3:**
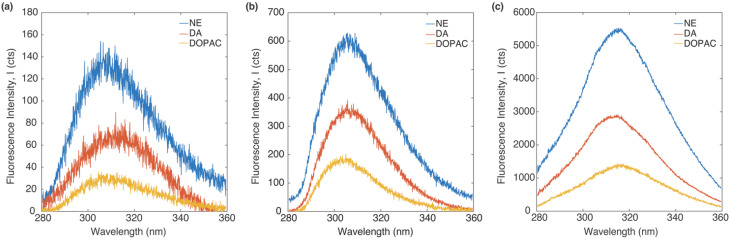
Fluorescence emission spectrum of DA, DOPAC and NE in a PVA thin film spin-coated on (a) a silicon, (b) an Al 30 nm thin film, and (c) a P300, after exposure to a 266 nm CW laser for 0.5 second.

Fig. 3 shows the emission spectrum of DA, NE and DOPAC on a silicon wafer, an Al 30 nm thin film, and a P300 Al hole array substrate with an integration time of 0.5 second. All molecules were dissolved in 0.25 wt% PVA with 1 mM concentration. The excitation source is a 266 nm CW laser. All fluorescence spectrum shows an emission peak between 300 and 320 nm. The peak locations for molecules on the P300 Al hole array are red-shifted compared to those on the silicon and the Al 30 nm thin film. The red-shift of the emission peaks can be attributed to the change in the local refractive index on the Al hole array substrate.^46–48^ The intensity of the emission spectrum was the highest for NE, followed by DA and DOPAC, on all three types of sample substrates, as well as in the bulk solution. The substrates strongly affect the native fluorescence intensity. The fluorescence intensity on a P300 Al hole array substrate is the highest, followed by an Al 30 nm thin film and a silicon substrate. This observed fluorescence signal enhancement is due to the surface plasmon resonance of the P300 Al hole array.^39^

**Fig. 4:**
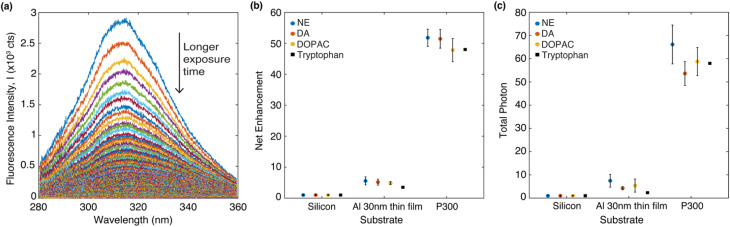
(a) DA emission spectrum overtime on a P300 Al hole array. The arrow points to the direction of time evolution. (b) Net enhancement of four different molecules in PVA deposited on three different types of substrates. (c) Total photon enhancement of four different molecules in PVA deposited on three different types of substrates. The data on tryptophan are from our previous study.^39^ In (b) and (c), the data represent the averages of five measurements, each taken from a different spot on the sample. The error bars shown in (b) and (c) represent the standard deviation of the five measurements.

Fig. 4a shows a representative set of the evolution of the DA fluorescence spectrum (on a P300 Al hole array) over time with an acquisition time of 0.5 second per spectrum. The native fluorescence completely bleaches to the background noise after 90 seconds. From the fluorescence decay series, the fluorescence net enhancement per excitation cycle and the total photon yield can be calculated.^39^ The procedure to calculate fluorescence net enhancement and total photon yield is described below. The fluorescence decay series of DA, NE and DOPAC on three different substrates (silicon, Al 30 nm thin film, P300 Al hole array) can be found in Fig. S2 in the ESI.†

The fluorescence decay series was measured for 90 seconds continuously with 0.5 second integration time per spectrum and it shows that the fluorescence intensity reduces over time while the peak positions are stable. We denote the intensity as *I*(*λ*,*t*), which is wavelength and time-dependent. For analysis of photobleaching rate, we indicate *S*(*t*), which integrated fluorescence intensity over the wavelength range 280 to 360 nm and can be written as 
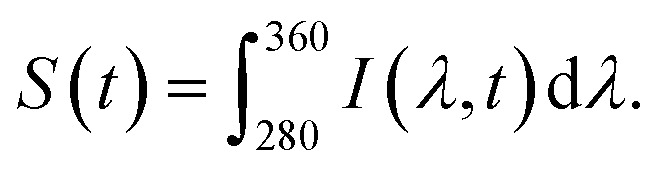
 Net enhancement is calculated by taking the ratio of *S*(*t* = 0.5) of each molecule on an Al 30 nm thin film and a P300 Al hole array to that on a silicon substrate. Fig. 4b shows the net enhancement of molecules on an Al 30 nm thin film and a P300 Al hole array. The P300 Al hole array substrate has the highest enhancement of all three substrates, with 51× enhancement for DA, 52× for NE, and 48× for DOPAC. The Al 30 nm thin film substrate shows a smaller enhancement, with 5.2× enhancement for DA, 5.6× for NE, and 4.8× for DOPAC. The total number of photons collected from the molecule before fully photobleaching is calculated by integrating *S*(*t*) from time 0 second (*t* = 0) to 90 second (*t* = 90). Total photon yield enhancement is calculated by taking the ratio of total photons emitted by molecules on an Al 30 nm thin film and a P300 Al hole array to that on a silicon substrate. The P300 Al hole array substrate shows a total photon yield enhancement of 54× for DA, 66× for NE, and 59× for DOPAC, and the Al 30 nm thin film shows a total photon yield enhancement of 4.2× for DA, 7.5× for NE, and 5.4× for DOPAC (Fig. 4c). The net enhancement and total photons reported in Fig. 4b and c are the averages of five measurements, with each measurement taken from a different spot on the sample. The error bars shown in Fig. 4b and c represent the standard deviation of the five measurements. For comparison, the net enhancement and total photon yield enhancement values for tryptophan on an Al hole array with 300 nm hole spacing from a previous study^39^ are also marked in Fig. 4b and c. The net enhancement and total photon yield enhancement values are very similar for all four different molecules.

**Fig. 5:**
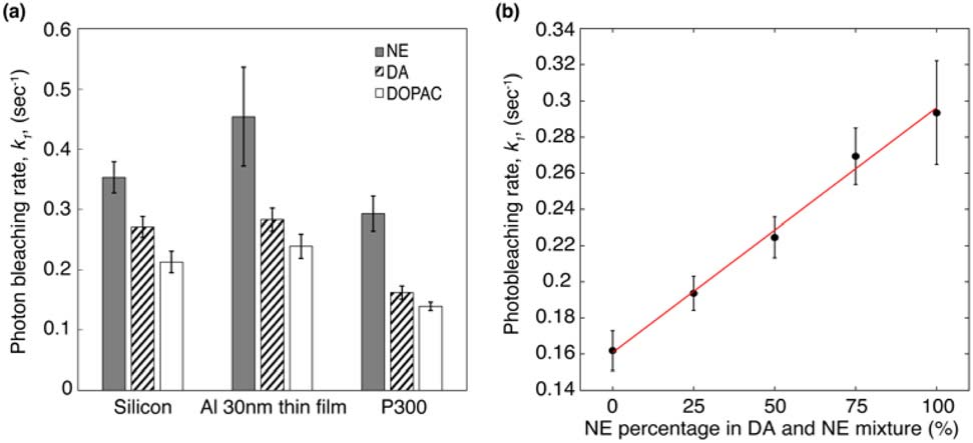
(a) The average fitted photon dominant photon bleaching rates (*k*_1_) of different three molecules deposited on silicon, Al 30 nm thin film, and P300 Al hole array substrates. The data is the average photon bleaching rates calculated from five measurements on each substrate. (b) The dominant photobleaching (*k*_1_) rate of NE and DA mixture deposited on a P300 Al hole array. The error bars shown in (a) and (b) represent the standard deviation of the five measurements.

From the fluorescence decay series, the photon bleaching rates of molecules can be obtained by fitting *S*(*t*) with a two-term exponential function *S*(*t*) = *a* × exp(*k*_1_*t*) + *b* × exp(*k*_2_*t*). The first term *k*_1_ is the fast decay rate, and the second term *k*_2_ is the slow decay rate.^39^ Biexponential photobleaching kinetics originates from the presence of a triplet state in the molecules.^49^ In ESI Fig. S3,† we presented the decay curves of each molecule deposited on three different substrates. Each plot represents the average of five measurements taken from five different spots on the sample. The error bars indicate the standard deviation of these five measurements. Fig. 5a shows the average photon bleaching rates *k*_1_ calculated from five measurements for each substrate. The fitted photon bleaching rates and the standard deviations from all measurements are listed in Table S1 in the ESI.† The fast decay rate is the dominant decay rate since the number of photons contributing to *k*_1_ is at least 3 times larger than *k*_2_, as evident from the fitted amplitudes in Table S2.† Therefore, we will only compare the dominant decay rates in the following discussion. Comparing the three different molecules, we found that: *k*_1_ of NE is the fastest of all three types of substrates and followed by DA, and DOPAC. The results indicate that DOPAC is the most photostable molecule, followed by DA and NE. The differences of *k*_1_ among different molecules are larger than the standard deviation calculated from five measurements. Our results indicate that photobleaching rates of native fluorescence are promising new mechanisms to differentiate molecules with similar structures.

Comparing the dominant photon bleaching rates *k*_1_ among the three substrates, we found that: for all three molecules, the Al 30 nm thin film substrate has the fastest *k*_1_, and the P300 Al hole array substrate has the slowest *k*_1_. For the P300 Al hole array, *k*_1_, the dominant photon bleaching rates of DA, NE and DOPAC are reduced by 1.7×, 1.5×, and 1.7× respectively, compared to the Al 30 nm thin film. The faster photon bleaching rate on the Al 30 nm thin film compared to the silicon substrates is likely due to the higher non-radiative rate of molecules near a metallic thin film. The slower photon bleaching rates on the P300 Al hole array are attributed to enhanced Purcell factor near the plasmonic substrates.^50^ Since DA and NE have a very similar molecular structures and almost identical absorption/emission, differentiating DA and NE in a mixture is a challenge. We will focus our discussions below on differentiating DA and NE using their photobleaching rates. From Fig. 5a, we calculated the differences in *k*_1_ for DA and NE to be 0.082 s^−1^, 0.171 s^−1^ and 0.13 s^−1^ on silicon, an Al 30 nm thin film and a P300 Al hole array. The differences in the dominate photobleaching rates for DA and NE were 2× and 1.6× larger on an Al 30 nm thin film and a P300 Al hole array than on a silicon substrate. Our results show that by depositing DA and NE onto metallic thin films or plasmonic hole arrays, the differences in their photon bleaching rate can be enlarged, in favor of selective detection of DA and NE.

In order to demonstrate that the photobleaching rates can be used to differentiate DA and NE, we measured the photobleaching rates of mixtures containing different concentrations of DA. DA and NE mixture were prepared in volume ratios of 1 : 0, 3 : 1, 1 : 1, 1 : 3, and 0 : 1 (100%, 75%, 50%, 25%, and 0% DA in mixtures). Fluorescence decay series of mixtures deposited on a P300 Al hole array was performed using an 0.5 second integration time for 90 seconds. Photobleaching rates of the mixture were obtained by fitting the decay series with a two-term exponential function. Fig. 5b shows the dominant photobleaching rate (*k*_1_) according to the mixing ratio. The scattered data points were fitted to a linear function. The dominant photobleaching rates increase almost linearly with the percentage of NE in the mixture. Our results indicate that in a mixture with unknown concentrations of NE and DA, the average photobleaching rates can potentially be used for determining the concentration of NE or DA in the mixture.

## Conclusions

In conclusion, we report the engineering of native fluorescence of neurotransmitters using metal thin film or nanostructure for the first time in the literature. We measured the native fluorescence of three monoamine neurotransmitters, dopamine (DA), norepinephrine (NE), and 3,4-dihydroxyphenylacetic acid (DOPAC) on a 30 nm-thick Al thin film and an Al hole array with 300 nm hole spacing. We observed an average of 50× net enhancement factor and an average of 60× total photon yield enhancement of the three molecules on the P300 Al hole array substrate. The Al hole array substrate not only enhanced the native fluorescence of neurotransmitters but also reduced their photobleaching rates. A 1.5 to 1.7× reduction in the dominate photon bleaching rate on a P300 Al hole array was observed compared to an Al 30 nm thin film. Net fluorescence enhancement and improved total photon yields are desirable for the highly sensitive detection of neurotransmitters using their native fluorescence. Other than sensitivity, selectivity is an important factor in the design of a biosensor. We propose the photobleaching rates of neurotransmitters as a new sensing mechanism to differentiate molecules with similar structures. Experimentally, we observed that the native fluorescence photobleaching rates of DA, NE and DOPAC are distinctively different from one another on a silicon substrate. The photobleaching rates of molecules can be engineered by metallic substrates as we observed that placing molecules near an Al thin film or hole array amplified the differences between the molecules. The tunability of the photobleaching rates can be employed to differentiate multiple neurotransmitters with similar structures in a mixture. To demonstrate the potential of using photobleaching rates to selectively detect neurotransmitters in a mixture, we mixed DA and NE at a different concentration ratio and we found out that the average photobleaching rates of the mixture is proportional to the concentration of NE in the mixture. Our findings present early evidence that plasmonic engineered native fluorescence has the potential to achieve sensitive and selective detection of neurotransmitters.

## Author contributions

Ji-Young Lee and Yunshan Wang contributed to the formulation and evolution of overarching research aims, writing and editing the manuscript. Ji-Young Lee conducted measurements, collected and analysed data for figures in the manuscript. Mohammad Mohammadi collected and analysed data to obtain quantum yields of the molecules.

## Conflicts of interest

There are no conflicts to declare.

## Supplementary Material

RA-013-D3RA05405E-s001
