# Supplementary material for: Detecting and differentiating neurotransmitters using ultraviolet plasmonic engineered native fluorescence
Source: RSC Adv. 2023 Nov 7;13(46):32582–8. doi: 10.1039/d3ra05405e (PMC10628848; doi:10.1039/d3ra05405e)
Supplement: RA-013-D3RA05405E-s001 [file RA-013-D3RA05405E-s001.pdf]

Electronic Supplementary Material (ESI) for RSC Advances.
This journal is © The Royal Society of Chemistry 2023

Supplementary Material

Detecting and differentiating neurotransmitters using Ultraviolet plasmonic engineered native fluorescence

Ji-Young Lee[a], Mohammad Mohammadi[a] and Yunshan Wang*[,a]

[a] Department of Chemical Engineering, University of Utah, Salt Lake City, 84112, USA
*Corresponding author: Yunshan Wang - yunshan.wang@utah.edu

Characterization of the aluminum hole-array

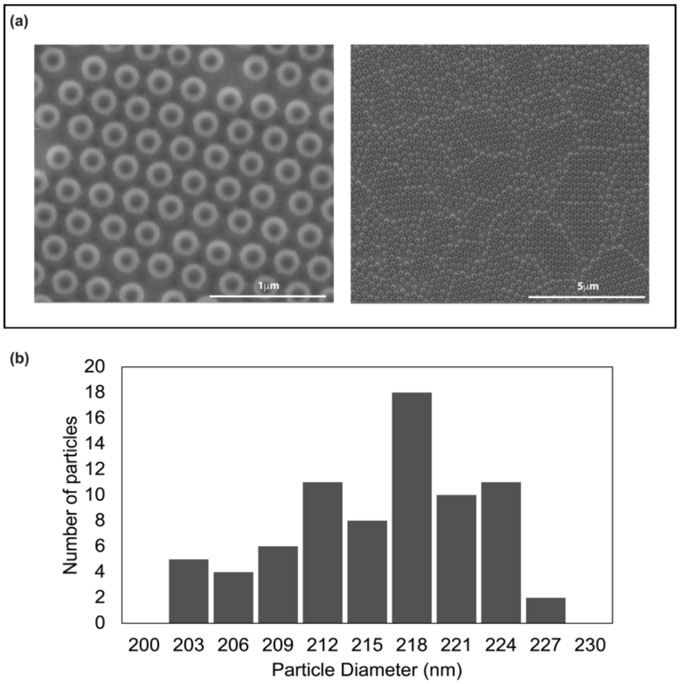

Fig. S1 (a) SEM images of the polystyrene (PS) particles following the etching process with different magnifications. (b) The size distribution of the etched PS particles was determined using SEM images and found to have an average diameter of 216 (±6) nm.

Native fluorescence decay series

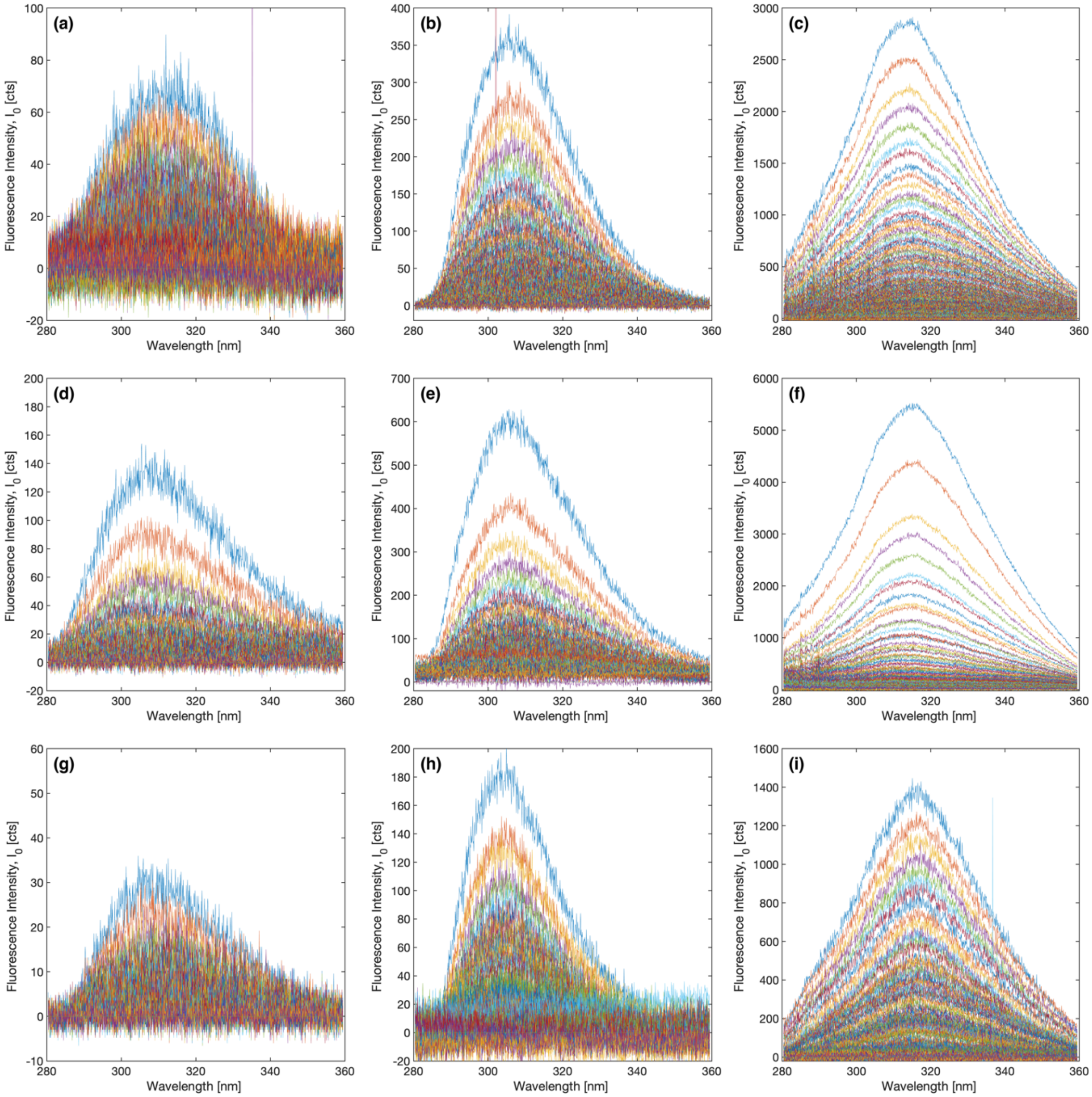

Fig. S2 Fluorescence photo decay series for DA deposited on (a) silicon, (b) an Al 30nm thin film and (c) a P300, NE on (d) silicon, (e) an Al 30nm thin film and (f) a P300, and DOPAC on (g) silicon, (h) an Al 30nm thin film and (i) a P300.

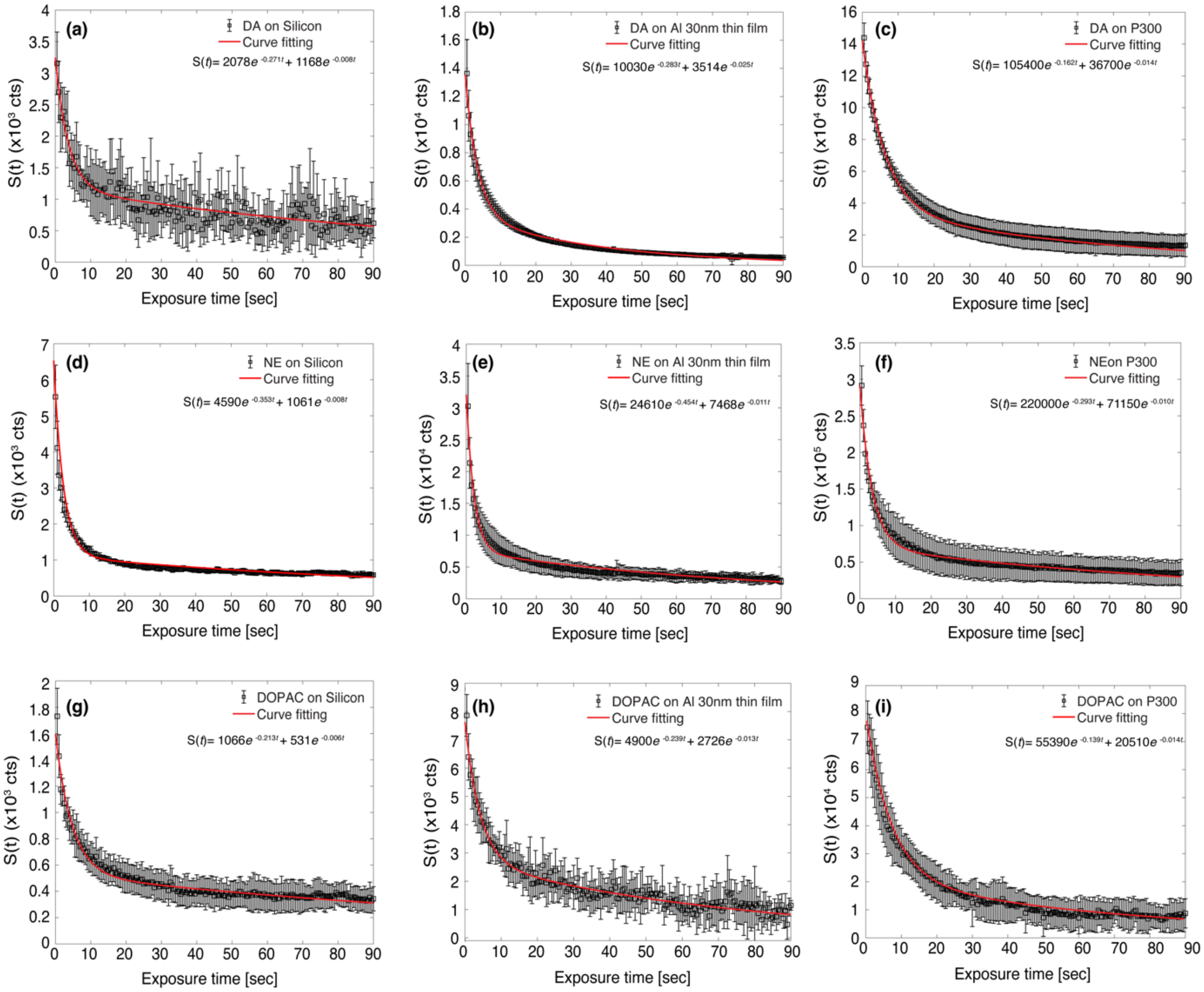

Fig. S3 The integrated fluorescence intensity S(t) versus exposure time for DA deposited on (a) silicon, (b) an Al 30nm thin film and (c) a P300, NE on (d) silicon, (e) an Al 30nm thin film and (f) a P300, and DOPAC on (g) silicon, (h) an Al 30nm thin film and (i) a P300. The solid red line is the two exponential curve fitting.

Table S1 The fitting decay rate k_1 and k_2 of the integrated fluorescence intensity S(t) of DA, NE and DOPAC deposited on silicon, Al 30nm thin film and P300. The decay rates are the two exponential curve fitting parameters fitted on every five different spots on the sample ($S(t) = a \times \exp(k_1 t) + b \times \exp(k_2 t)$).

	**Silicon**			**Al 30nm thin film**			**P300**		
	NE	DA	DOPAC	NE	DA	DOPAC	NE	DA	DOPAC
	0.335	0.246	0.185	0.360	0.317	0.240	0.342	0.153	0.135
	0.330	0.265	0.231	0.479	0.279	0.258	0.277	0.153	0.131
k_1	0.391	0.283	0.207	0.447	0.271	0.243	0.269	0.179	0.150
	0.341	0.272	0.218	0.408	0.274	0.247	0.291	0.157	0.138
	0.369	0.289	0.222	0.578	0.273	0.204	0.288	0.166	0.143
std (σ)	0.026	0.017	0.018	0.082	0.019	0.020	0.029	0.011	0.007
Average	**0.353**	**0.271**	**0.213**	**0.454**	**0.283**	**0.239**	**0.293**	**0.162**	**0.139**
	0.007	0.006	0.004	0.006	0.030	0.008	0.013	0.020	0.019
	0.007	0.003	0.010	0.014	0.026	0.017	0.008	0.020	0.012
k_2	0.009	0.005	0.005	0.004	0.025	0.022	0.009	0.009	0.021
	0.008	0.007	0.005	0.015	0.023	0.014	0.010	0.010	0.005
	0.009	0.019	0.005	0.019	0.023	0.006	0.009	0.011	0.014
Average	**0.008**	**0.008**	**0.006**	**0.011**	**0.025**	**0.013**	**0.010**	**0.014**	**0.014**

From Table S1, we calculated that the differences in k_1 between DA and NE are larger than 3 times the standard deviations (σ) of DA or NE's k_1 ($\Delta k_1 > 3\sigma$) on silicon and aluminum hole array. The differences in k_1 between DA and NE are larger than 2 times the standard deviations ($\Delta k_1 > 2\sigma$) on aluminum thin film. The differences in k_1 between NE and DOPAC are larger than 3 times the standard deviations ($\Delta k_1 > 3\sigma$) for all 3 substrates. The differences in k_1 between DA and DOPAC are larger than 3 times the standard deviations ($\Delta k_1 > 3\sigma$) for silicon, and 2 times the standard deviations ($\Delta k_1 > 2\sigma$) for aluminum thin film or aluminum hole array. We believe the differences in k_1 between neurotransmitters are significant.

Table S2 The fitting amplitude a and b of the integrated fluorescence intensity S(t) of DA, NE and DOPAC deposited on silicon, Al 30nm thin film and P300. The decay rates are the two exponential curve fitting parameters fitted on every five different spots on the sample ($S(t) = a \times \exp(k_1 t) + b \times \exp(k_2 t)$).

	**Silicon**			**Al 30nm thin film**			**P300**		
	NE	DA	DOPAC	NE	DA	DOPAC	NE	DA	DOPAC
	3534	3466	918.0	18825	13061	4294	238190	110600	51799
	3653	2325	1219	19402	10764	4648	206210	96971	51434
a	5665	1856	978	25581	9086	5126	228240	100560	39775
	4687	1071	1063	31625	8246	4789	233850	114690	74116
	5413	1668	1152	27630	8986	5646	193400	104170	59814
Average	**4590**	**2078**	**1066**	**24610**	**10030**	**4900**	**220000**	**105400**	**55390**
	998.4	361.7	460.9	6400	4555	1948	52640	36150	24882
	1042	1120	422.1	9202	3692	3569	99874	31688	10574
b	1141	1391	485.0	3467	3335	2390	60538	48365	30329
	1005	1096	574.8	9779	2788	3391	41265	35207	24293
	1120	1870	710.3	8491	3199	2334	101420	32109	12485
Average	**1061**	**1168**	**530.6**	**7468**	**3514**	**2726**	**71150**	**36700**	**20510**

Fluorescence Quantum yield

The quantum yield refers to the proportion of photons that are emitted compared to the number of photons that are absorbed[1]. The most common way of determining the quantum yield (Φ) of samples is the "Relative Method," which requires knowing the absorbance of both the reference and the sample solution and relies on using well-characterized reference standards with known Φ_R value and optical properties closely matching the sample of interest [1,2]. It compares the integrated fluorescence intensity of a sample of known Φ_R, generally referred to as the reference, against the samples with unknown Φ_S.

This method is only applicable to samples that can go into solution because the measurement requires knowledge of the refractive index of the solvent and the absorbance of both reference and sample[1]. It uses a conventional fluorescence spectrometer which detects only a fraction of the light emitted due to a wide range of factors, including the refractive index of the solvent, the scattering of light by the sample, the emission wavelength, the 90° arrangement of the excitation and emission optics.

The standard samples should be chosen to ensure they absorb at the excitation wavelength of choice for the test sample and, if possible, emit in a similar region to the test sample. The standard sample must be well-characterized and suitable for such use. The quantum yields of the known standard compounds are primarily independent of excitation wavelength[1]. Therefore, we used aqueous Tryptophan as a standard solution to measure the quantum yield (Φ_S) of DA, NE, and DOPAC dissolved in water at concentrations of 0, 10, 20, 30, 40, 50 micromolar using the following formula:

$$\Phi_S = \Phi_R \frac{I}{I_R} \frac{OD_R}{OD} \frac{n^2}{n_R^2} = \Phi_R \frac{(I/OD)_{sample}}{(I_R/OD_R)_{Ref}} \frac{n^2}{n_R^2} \qquad (1)$$

where Φ is the quantum yield, I is the integrated intensity, OD is the optical density, and n is the refractive index. The subscript R refers to the reference fluorophore of known quantum yield. Optical density (OD) is related to the speed of light through the medium and takes refraction into account, but absorbance (A) does not take the refraction of light into account and only considers the amount of light lost. We can ignore the differences between OD and A since the refraction of light is negligible. The following equation can be used to calculate A or OD from transmittance (T).

$$Absorbance(A) = Optical\ Density(OD) = -\log_{10} Transmittance(T) \rightarrow T = 10^{-OD} = 10^{-A} \quad (2)$$

The ratio of integrated intensity per Optical Density can be obtained by the plot of integrated fluorescence intensity versus absorbance. In order to do so, Firstly, the absorbance at the excitation wavelength of samples in 6 different concentrations was recorded using a UV-vis spectrometer. Then, the fluorescence intensity of samples was also recorded using Fluorometer. After calculating the integrated fluorescence intensity, a graph of integrated fluorescence intensity versus absorbance can be plotted. The fluorescence intensity of samples and the standard were measured with identical spectrometer settings such as excitation wavelength, slit widths of excitation and emission monochromator, scan speed, and integration time[3]. The linear regression of data points gives a straight line with the slope of m; this gradient is equal to the ratio of I/OD; therefore, equation (1) can be rewritten like below:

$$\Phi_S = \Phi_R \frac{m_{sample}}{m_{Ref}} \left(\frac{n}{n_R}\right)^2 \quad (3)$$

Having used dilute concentrations in micromolar ranges of DA, DOPAC, and NE dissolved in DI water and also choosing Tryptophan in DI water as the reference standard, there would be no significant change in refractive indexes because the solvent used in the samples and the standard solution is the same. Fig. 2c in the main article, plots the integrated fluorescence intensity of DA, DOPAC and NE for each concentration and their absorbance at the excitation wavelength. The reason for choosing the range of 0 to 50 μM concentration for the compounds was to minimize re-absorption effects[4]. Absorbances in the 10 mm fluorescence cuvette should never exceed 0.1 at and above the excitation wavelength. Above this level, non-linear effects may be observed due to inner filter effects, and the resulting quantum yield values may be perturbed.

Using the quantum yield value of 13% for Tryptophan in water[1], one of the good fluorescent standard solutions, available in literatures, the quantum yield of the three tested substances including DA, DOPAC, and NE was reported in Table S3. Propagation of error is used to estimate the uncertainty in quantum yield calculation for each component. The propagated uncertainty associated with the quantum yield values (±0.0080 for DA, ±0.0021 for DOPAC and ±0.0062 for NE) represent the standard errors[5] in the measurements and reflect the precision of the experimental data.

Table S3 Quantum yield results for DA, DOPAC, NE

Component	Φ
Tryptophan	0.13 ± 0.01 [1]
DA	0.0593 ± 0.0080
DOPAC	0.0167 ± 0.0021
NE	0.0634 ± 0.0062

References

1. J. R. Lakowicz, *Principles of fluorescence spectroscopy*, Springer, 2006.
2. B. Xiong, Y. Chen, Y. Shu, B. Shen, H. N. Chan, Y. Chen, J. Zhou and H. Wu, *Chemical Communications*, 2014, **50**, 13578-13580.
3. C. Würth, M. Grabolle, J. Pauli, M. Spieles and U. Resch-Genger, *Nature Protocols*, 2013, **8**, 1535-1550.
4. S. Dhami, A. J. D. Mello, G. Rumbles, S. M. Bishop, D. Phillips and A. Beeby, *Photochemistry and Photobiology*, 1995, **61**, 341-346.
5. J. Taylor, *Introduction to error analysis, the study of uncertainties in physical measurements*, 1997.
